# Tranexamic acid for control of blood loss in bilateral total knee replacement in a single stage

**DOI:** 10.4103/0019-5413.77135

**Published:** 2011

**Authors:** Mandeep S Dhillon, Kamal Bali, Sharad Prabhakar

**Affiliations:** Department of Orthopedics, Postgraduate Institute of Medical Education and Research (PGIMER), Chandigarh, India

**Keywords:** Antifibrinolytic, blood loss, hemoglobin, knee arthroplasty, tranexamic acid

## Abstract

**Background:**

Tranexamic acid (TEA) reduces blood loss and red cell transfusions in patients undergoing unilateral total knee arthroplasty (TKA). However, there is not much literature regarding the use of TEA in patients undergoing bilateral TKA in a single stage and the protocols for administration of TEA in such patients are ill-defined.

**Materials and Methods:**

We carried out a case control study evaluating the effect of TEA on postoperative hemoglobin (Hb), total drain output, and number of blood units transfused in 52 patients undergoing bilateral TKA in a single stage, and compared it with 56 matched controls who did not receive TEA. Two doses of TEA were administered in doses of 10 mg / kg each (slow intravenous (IV) infusion), with the first dose given just before tourniquet release of the first knee and the second dose three hours after the first one.

**Results:**

A statistically significant reduction in the total drain output and requirement of allogenic blood transfusion in cases who received TEA, as compared to the controls was observed. The postoperative Hb and Hb at the time of discharge were found to be lower in the control group, and this result was found to be statistically significant.

**Conclusion:**

TEA administered in patients undergoing single stage bilateral TKA helped reduce total blood loss and decreased allogenic blood transfusion requirements. This might be particularly relevant, where facilities such as autologous reinfusion might not be available.

## INTRODUCTION

Total knee arthroplasty (TKA) is usually associated with marked postoperative blood loss.^1^ The use of a pneumatic tourniquet ensures a dry surgical field and minimal intraoperative bleeding, but it augments fibrinolysis stimulated by surgical trauma.^2^–^7^ This activation of the fibrinolytic system might lead to high postoperative blood loss and this could be more significant in patients undergoing bilateral TKA in a single stage. The increased requirement of blood transfusion predisposes these patients to high postoperative morbidities. This is especially true in developing countries where allogenic blood transfusion is the only option available, due to the absence of facilities of intraoperative blood harvest and autologous retransfusion in most of the centers.

Tranexamic acid (TEA) is a synthetic analog of the amino acid lysine and inhibits fibrinolysis locally, without any effect on the fibrinolysis in the plasma from peripheral venous circulation.^8^ Multiple previous studies have shown a reduction in blood loss and postoperative transfusion requirements with the use of TEA in patients undergoing unilateral total knee arthroplasty.^8^–^15^ However, there is limited literature^16^ regarding the use of TEA in patients undergoing concurrent bilateral TKA. These are the patients expected to be associated with higher transfusion requirement and thus more likely to benefit from such antifibrinolytic therapy.

We evaluated the effect of the use of TEA on postoperative blood loss and allogenic transfusion requirements in patients undergoing bilateral TKA in a single stage. We worked on the hypothesis that administration of TEA to patients undergoing bilateral TKA in a single stage would help reduce postoperative blood loss and decrease allogenic blood transfusion requirements. This would in turn help reduce postoperative morbidities, which usually occur in as a result of massive allogenic blood transfusions. We also evaluated the safety of TEA administration to patients undergoing simultaneous bilateral TKA.

## MATERIALS AND METHODS

The present investigation was a case control study started in from February 2008 to January 2010. We studied all the patients (52 patients) with primary osteoarthritis of the knee undergoing simultaneous bilateral TKA who had been administered TEA as per our protocol. Patients with a documented history of thromboembolic disease, cerebrovascular disease, recent myocardial infarction or unstable angina, a coagulation defect, chronic renal or liver disease, and those with allergy to TEA were excluded from the study. As we excluded the patients with more than usual risk for deep venous thrombosis (DVT) from our study, we did not attempt to preoperatively screen the patients included in our study for DVT. As a control group we obtained the data of all the patients (56 patients) with primary osteoarthritis of the knee operated with single staged bilateral TKA, for over a period of two years, starting from January 2006 to December 2007, in the department. For the study group, our protocol included administration of one intraoperative dose of 10 mg per kg of TEA (given as infusion over 10 minutes) approximately 10 minutes before deflation of the first tourniquet (at the time of cementing of the first knee), and the second dose (also over 10 minutes) three hours after the first. All the surgeries were performed by the senior author (MSD), using a surgical technique standardized to the same design cemented knee prosthesis (DePuy; Smith, and Nephew). A tourniquet and a bone plug (to block the femoral medullary cavity after the femoral cuts) were always used to decrease the blood loss. Closure was performed only after adequate hemostasis was obtained. An intra-articular negative suction drain was used in all the cases to measure postoperative blood loss; however, we did not attempt to measure the intraoperative blood loss. As a rule, the more clinically painful knee was operated first.

Preoperative investigations included hemoglobin (Hb), hematocrit (Hct), and a complete coagulogram. Postoperative Hb levels and Hct were measured six hours after surgery and at the time of discharge (usually after suture removal). A negative suction drain was kept for 48 hours and the drain output was recorded for day 0 and day 1. Allogenic blood transfusion in the form of whole blood was given to all patients with postoperative Hb less than 9 gm / dl and in patients with an Hct less than 28%. The transfusion requirement was also assessed on the basis of total drain output so as to replace the blood lost in the negative suction drain.

All the patients received thromboprophylaxis in the form of subcutaneous low molecular weight heparin starting from day 1 and continued till the time of discharge (14 days postoperatively in our setting). Postoperative use of DVT stockings, ankle pumps, and early mobilization was also ensured as a part of thromboprophylaxis.

Statistical analysis was carried out with SPSS software version 16. On analysis, using one sample Kolmogorov-Smirnov Test, all the variables (except preoperative and postoperative Hct) were found to have a normal distribution of data. Therefore, parameter tests of significance (independent t-test) were used for statistical analysis. Analysis of data pertaining to Hct values was done using nonparametric tests (Mann-Whitney U test). Power analysis was also done and a *P* value less than 0.05 was taken as statistically significant.

## RESULTS

The cases included 52 patients who received TEA during concurrent bilateral TKA, while the controls included 56 patients without TEA administration. The cases and controls were found to be both age and sex matched on statistical analysis [Table 1].

**Table 1:** Patient characteristics of the two groups along with their age and weight range

	TEA group	Controls	*P* value
No. of subjects	52	56	
Sex (males / females)	18 / 34	20 / 36	
Male to female ratio	0.53	0.55	0.905#
Mean age (years) with range	65.75 (50 − 82)	67.25 (51 − 82)	0.359
Mean weight (kg) with range	74 (60 − 94)	77 (62 − 106)	0.194

# Chi-Square Test

The mean preoperative Hb and both pre- and postoperative Hct values were found to be similar in both groups [Table 2]. However, the postoperative Hb was found to be lower in the control group as compared to the TEA group and this difference was found to be statistically significant (*P* = 0.000). The power of this test was found to be high; approaching 1.00 (0.996). Although Hb at discharge was found to be lower for the control group as compared to the TEA group, this result was not found to be statistically significant (*P* = 0.075).

**Table 2:** Mean Hb (g / dl) and Hct (%) along with the standard deviations (± SD) before and after surgery

	TEA group	Controls	*P* value	Power
Preoperative Hb	12.78 ± 1.85	13.04 ± 1.72	0.459	0.119
Preopearative Hct	35.30 ± 2.94	35.78 ± 2.26	0.349	0.147#
Postoperative Hb (six hours after surgery)	11.79 ± 1.96	10.25 ± 1.40	0.000*	0.996
Postoperative Hct	36.61 ± 2.16	36.89 ± 2.47	0.536	0.117#
Hb at discharge	12.26 ± 1.45	11.78 ± 1.33	0.075	0.436

* Significant

# Mann-Whitney U Test

The total postoperative drain output [Table 3] was found to be lower in patients who received TEA as compared to the control group (275 ml vs. 810 ml) and this relation was also found to be statistically significant (*P* = 0.000). The sample size was large enough for this conclusion to be made (power 1.00 in all). This implied a decrease in total blood loss in patients who were administered TEA during bilateral TKA. As such the amount of allogenic blood transfusion (BT) requirements [Table 3] were also reduced in these patients as compared to the control group (0,80 units vs. 3.17 units). This decrease in transfusion requirement in TEA group patients was also found to be statistically significant (*P* = 0.000). Administration of TEA thus helped us to avoid any form of transfusion in 25 of the 52 patients in the TEA group, while all the patients in the control group had required some amount of blood transfusion [Table 4].

**Table 3:** Mean drain output and transfusion requirements along with standard deviations (± SD)

	TEA group	Controls	*P* value	Power
Drain output on day 0 (ml)	204.81 ± 102.90	577.86 ± 168.16	0.000*	1.00
Drain output on day 1 (ml)	69.80 ± 34.14	231.79 ± 87.26	0.000*	1.00
Total drain output (ml)	274.62 ± 128.34	809.64 ± 227.30	0.000*	1.00
Units of blood transfused	0.80 ± 0.90	3.17 ± 0.81	0.000*	1.00

* Significant

**Table 4:** Comparison of TEA and Control group in relation to need of blood transfusion

	BT needed	BT not needed	Total
Control group	56	0	56
TEA group	27	25	52
Total	83	25	108

BT = Blood transfusion

No adverse effects like nausea, vomiting, diarrhea or hypersensitivity were found in any of the patients receiving TEA. Superficial infection requiring an extended course of antibiotics developed in five knees of our patient population (three in the TEA group and two in the control group). Barring one patient in the control group, none of the other patients developed deep infection requiring repeated debridement. The deep infection that developed in one of our patients in the control group ultimately required implant removal and knee arthrodesis.

Postoperative wound soakage developed in one of the knees in four of our patients (two in each group). No patient had soakage in both the knees. However the soakage was found to be minimal and settled in a day or two in all the patients. One patient in the control group had developed signs and symptoms of deep venous thrombosis. She was managed by therapeutic dosage of low molecular weight heparins. No such episode was seen in the TEA group. Pulmonary embolism was not seen in any of our patients.

## DISCUSSION

Total knee arthroplasty is usually associated with the average reported postoperative blood loss in unilateral TKA ranging from 761 ml to 1784 ml.^10^,^17^–^22^ This blood loss is expected to be higher in bilateral TKA in a single stage, and as such, the requirements for blood transfusion increases for these patients. Preoperative autologous blood transfusion with or without erythropoeitin and intraoperative blood salvage using ‘cell savers’ are ways to decrease the requirements of allogenic blood transfusion. However, these methods of autologous transfusion are rarely used in developing countries. Thus, demand for allogenic blood transfusion becomes really high in patients undergoing bilateral TKA at a single stage.

Various antifibrinolytic agents such as, aprotinin, e-aminocaproic acid, and TEA can help reduce blood loss in TKA. Of these TEA is preferred as it is cheaper and less allergenic than aprotinin and is more potent than e-aminocaproic acid. TEA acts by competitively inhibiting the activation of plasminogen to plasmin. It also blocks the binding of plasminogen to fibrin, which retards fibrinolysis, thus reducing blood loss by clot stabilization rather than promotion of clot formation.^23^

Previous studies^24^ have demonstrated a minimum dosage of 10 mg / kg of TEA, to obtain the desired antihemorrhagic effect. Hence, we also used the same dosage in our investigation so as to minimize the side effects. As the time from cementing to release of the tourniquet is fairly constant and mostly dependent on the cement curing time, we felt this was a reliable time to administer the drug in all the cases. Considering that the mean duration of the effect of TEA is around three hours, we repeated the dose after this period, to prolong the effect of the drug to the first six hours, when most bleeding is expected to occur. Although prolonged stasis could potentially increase the risk of thrombosis, we did not note an increased incidence of thrombosis in the extremity operated second, which had received the first dose of TEA prior to tourniquet inflation. Various clinical trials have also shown no increase in thromboembolic complications when antifibrinolytics are given before tourniquet inflation.^20^,^24^ We had not routinely performed compression ultrasonography or a Doppler-Echo to rule out DVT, which is a potential limitation of our study. Nevertheless, no clinically relevant thromboembolic episodes were found in our cases, which was consistent with the results of the earlier studies.^17^–^21^,^25^–^27^ We thus believe that the protocol adopted by us, using minimally effective dosage, was safe as far as thromboembolic complications are concerned. Whether TEA increases thromboembolic events in patients with pre-existing comorbidities (such as hypercoaguable stages, previous history of DVT, etc.) is still to be established. However, we believe that TEA should be preferably avoided in such a patient population.

Another limitation of our study was that we did not attempt to evaluate the intraoperative blood loss in the two groups. We only compared the postoperative blood loss by measuring the drain output. We thus could not exactly calculate the total blood loss in the two groups. This could explain the possible difference in the average blood loss measured by us (275 ml) as compared to another study^16^ by MacGillivray *et al*. (678 ml), using the same protocol of TEA administration, in simultaneous bilateral TKA. These authors^16^ had measured the intraoperative blood loss using special intra-articular drains, which we never attempted to use. Such a use of an intraoperative drain system definitely helps determine the exact blood loss during the surgery. Nevertheless, we had all the records of preoperative and postoperative Hb of the cases and the controls, which gave us a fairly decent idea about the total blood loss in the two groups in our study. The postoperative Hb showed a statistically significant fall in the control group as compared to the cases receiving TEA, thus implying a positive impact of TEA administration on blood loss. Also the total drain output was significantly lower in the TEA group, again confirming the usefulness of TEA as far as controlling blood loss during concurrent TKA is concerned.

One finding that we could not possibly explain was the decrease in drain output on day 1. TEA given by our protocol is believed to be most effective over the first six hours. However, the fact that the drain output was reduced up to 48 hours after the administration of TEA makes us believe that some residual effect of TEA might persist even after six hours of administration and further dose administration might be not needed even in cases of concurrent bilateral TKA.

The fact that all the patients in both the groups were operated by the same senior surgeon excluded the possibility of surgeon dependent factors affecting the results and in turn added strength to our study. The large sample size of our study also imparts strength to our statistics and we could use parametric tests to analyze our results. However, one potential weakness that remains is the case control nature of the study. As a result we were unable to evaluate some important factors in our study. For example comparison of total blood loss was not possible with our study design. This is because we did not have the records of intraoperative blood loss of the control group patients who were operated before this study was planned. A prospective randomized control trial type of study design would have overcome this limitation and further added strength to the interpretation.

Another strong point of the study was evaluation of the efficacy of TEA in patients with concurrent bilateral TKA. To date most of the studies have been done in case of unilateral TKA, for which TEA administration protocols have been well defined. No specific protocols exist for administration of TEA in bilateral TKA done at a single stage. To our knowledge there is only one study^16^ regarding use of TEA in concurrent bilateral TKA. The authors compared the effects of two-dosage regimens of TEA (10 mg/kg and 15 mg/kg) on blood loss and transfusion requirement with that of saline placebo in 60 patients undergoing concurrent bilateral TKA, with additional reinfusion autotransfusion from intra-articular drains. Mean blood loss was 462 mL in the 15 mL / kg group, 678 mL in the 10 mg/kg group, and 918 mL in the controls. Blood available for autotransfusion was greatest in the controls and least in the group with a dosage of 15 mg/kg. Combined autologous and allogenic transfusion volumes were similar in the treatment groups and significantly less than those in the controls. The authors concluded that with the use of an autologous reinfusion strategy, a lower dose is sufficient to lead to a lesser allogenic transfusion requirement.

Reinfusion autotransfusion, although an excellent modality, is not available at most of the centers in our country. Thus, simultaneous bilateral TKA is usually associated with massive allogenic blood transfusion requirements. This may in turn predispose to various complications like electrolyte imbalance, deranged coagulation, and hypersensitivity reactions in already compromised patients. We have seen in our study that the use of TEA reduces the average blood transfusion requirements from 3.17 units (in controls without TEA administration) to 0.80 units (in cases with TEA administration). We can thus conclude that TEA use might be a good solution to the problem of massive allogenic transfusion requirements especially in developing countries like India, where facilities of reinfusion autotransfusion might not be available.
